# Genetic and molecular landscape of comorbidities in people living with HIV

**DOI:** 10.1038/s41591-025-03887-1

**Published:** 2025-08-20

**Authors:** Javier Botey-Bataller, Nienke van Unen, Marc Blaauw, Willem A. J. W. Vos, Louise van Eekeren, Nadira Vadaq, Vasiliki Matzaraki, Annelies Verbon, Albert L. Groenendijk, Jéssica C. dos Santos, Maartje C. P. Cleophas, Janneke E. Stalenhoef, Marvin A. H. Berrevoets, Xun Jiang, Manoj K. Gupta, Nhan Nguyen, Cheng-Jian Xu, Leo A. B. Joosten, Mihai G. Netea, André J. A. M. van der Ven, Yang Li

**Affiliations:** 1 Department of Computational Biology for Individualised Infection Medicine, Centre for Individualised Infection Medicine, a joint venture between the Hannover Medical School and the Helmholtz Centre for Infection Research, Hannover, Germany; 2 TWINCORE, Centre for Experimental and Clinical Infection Research, a joint venture between the Hannover Medical School and the Helmholtz Centre for Infection Research, Hannover, Germany; 3 Department of Internal Medicine and Radboudumc Community for Infectious Diseases, Radboud University Medical Center, Nijmegen, The Netherlands; 4 Cluster of Excellence Resolving Infection Susceptibility (RESIST; EXC 2155), Hannover Medical School, Hannover, Germany; 5 Department of Internal Medicine and Department of Medical Microbiology and Infectious Diseases, Erasmus Medical Center, Erasmus University, Rotterdam, The Netherlands; 6 Department of Internal Medicine, OLVG Hospital, Amsterdam, The Netherlands; 7 Department of Internal Medicine, Elisabeth-Tweesteden Hospital, Tilburg, The Netherlands; 8 Department of Medical Genetics, Iuliu Hatieganu University of Medicine and Pharmacy, Cluj-Napoca, Romania; 9 Department of Immunology and Metabolism, Life and Medical Sciences Institute, University of Bonn, Bonn, Germany; 10 Lower Saxony Center for Artificial Intelligence and Causal Methods in Medicine (CAIMed), Hannover, Germany

**Keywords:** Epidemiology, HIV infections

## Abstract

People living with HIV (PLHIV) have an increased susceptibility to non-AIDS comorbidities. In this study, we systematically profiled 1,342 PLHIV across five omics layers and immune function. We found latent factors, resulting from integrating epigenomics, transcriptomics, proteomics, metabolomics and immune responses, linked to cardiovascular diseases, the presence of carotid plaque and chronic obstructive pulmonary disease in PLHIV. Mapping four omics layers to genetic variation identified 5,962 molecular quantitative trait loci, illustrating a common genetic regulation in PLHIV compared to healthy individuals. By performing Mendelian randomization, we uncovered host genetic-driven changes in baseline molecules causally related to immune responses upon stimulation with inactivated pathogens. Lastly, we uncovered that the inflammasome, genetically regulated by the *NLRP12* locus, contributes to systemic inflammation across multiple molecular layers. This study offers a unique catalog of genetic and molecular determinants of immune function in PLHIV and elucidates molecular pathways driving inter-individual variation in immune response and comorbidities.

## Abstract (web summary)

An integrated dataset combining genetics, epigenomics, transcriptomics, proteomics and metabolomics from 1,342 people living with HIV illuminates molecular pathways driving immune responses and comorbidities in this population.

## Main

PLHIV receiving antiretroviral therapy (ART) mostly restore their CD4^+^ T cell counts, protecting them from developing AIDS-related complications^1^. However, they still experience increased systemic inflammation and premature aging, which makes them prone to non-AIDS comorbidities, such as cardiovascular disease (CVD), that lower their quality of life^2^. There is a significant inter-individual variability in the susceptibility to comorbidities^3^, systemic inflammation^4,5^ and immune responses in PLHIV using ART, which may be caused by HIV-related factors, such as HIV duration, CD4 nadir and latest CD4 levels, as well as HIV-independent factors, such as age, genetics and concurrent infections^6^. The molecular mechanisms that drive this state and result in non-AIDS comorbidities are yet to be described.

Characterizing the inter-individual variation among PLHIV involves multiple molecular layers. Previous studies focused on profiling PLHIV at a single omics type, specifically looking into the proteomic^7^, metabolomic^8,9^ or epigenomic^10^ factors. In addition, host genetic variation also modulates disease outcome and progression in PLHIV^11^. By using statistical methods such as Mendelian randomization, genetic variants can be used as instruments to infer causal relationships between factors, allowing the identification of modulators that can serve as potential drug targets^12^. Such an approach has not yet been employed to investigate the pathophysiology of HIV infection at various omics layers^13^.

In the present study, we generated multi-omics and immune function profiles of 1,342 virally suppressed PLHIV of European ancestry. We applied three integrative approaches to decipher the drivers of inter-individual variation in PLHIV: multi-omics factor analysis (MOFA)^14^, quantitative trait locus (QTL) mapping and Mendelian randomization. By applying MOFA, we identified 21 latent factors (LFs) resulting from the combination of different omics. We performed QTL mapping for in vivo gene expression, protein and metabolite abundance and ex vivo cytokine production. Comparing our results with individuals without HIV revealed an overall concordance of genetic effects compared to those without HIV. Using Mendelian randomization, we found the circulating transcriptomic, proteomic and metabolomic programs causally linked to cytokine production capacity upon ex vivo stimulation of immune cells with inactivated pathogens. In addition, we identified the *NLRP12* locus and the inflammasome as crucial modulators of systemic inflammation in PLHIV, impacting carotid plaque formation. Our findings are publicly available through a web tool, available at https://lab-li.ciim-hannover.de/apps/hiv_xqtl_atlas/.

## Results

### Comprehensive multi-omics profiling of PLHIV

We performed a systems immunology study in 1,342 virally suppressed PLHIV, part of the 2000HIV Human Functional Genomics Partnership Program (2000HIV) study^15^. The cohort was split into a discovery cohort and a validation cohort: samples from three centers (*N* = 1,075) served as the discovery cohort, whereas samples from one center (*N* = 267) were used as the replication cohort. Overall, study participants were mostly male (89%), with a median age of 54 years, and had been living with HIV for a median of 10 years (Table 1).

**Table 1:** 2000HIV cohort overview

	Discovery	Validation
Sex		
Female	139 (11.24%)	30 (10.4%)
Male	1,098 (88.76%)	266 (89.86%)
Age (years)		
Median (min, max)	54 (19, 84)	53.5 (26, 77)
BMI (kg m^−2^)		
Median (min, max)	24.8 (15.5, 48.4)	25.4 (17.6, 40.4)
CD4 latest (cells per μl)		
Median (min, max)	0.7 (0.1, 3.2)	0.7 (0.1, 1.7)
HIV duration (years)		
Median (min, max)	13.1 (0.5, 42)	10.2 (0.5, 37)
Rapid progressors		
Yes/No/Missing	61/705/471	10/144/142

**Fig. 1:**
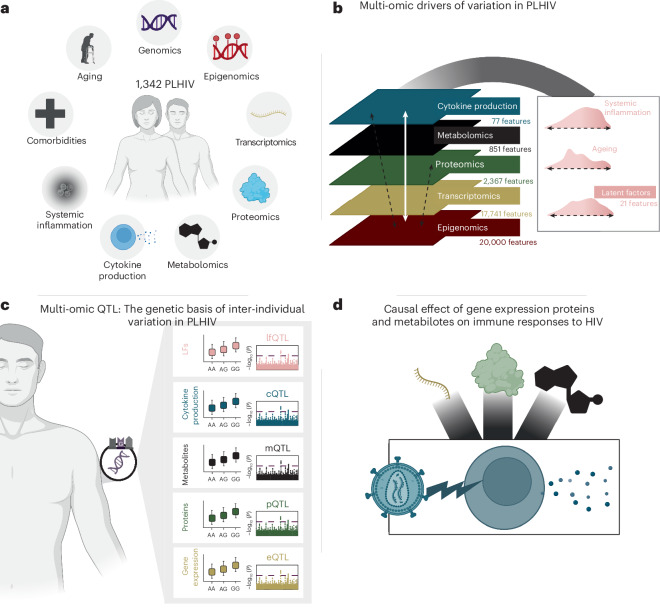
Integrative omics in PLHIV. **a**, Overview of the data available for the cohort. Multi-omics data, together with immune response profiling, were collected. Clinical data on non-AIDS comorbidities were available. **b**, Multi-omics integration. Features across all layers were integrated using MOFA. This captured LFs related to processes such as systemic inflammation and aging. **c**, Multi-omics QTL to dissect the genetic basis of inter-individual variation in PLHIV. **d**, Mendelian randomization to study the causal effect of circulating molecules on immune function in PLHIV. Schematics were created with BioRender.com. LF, latent factor.

Aiming to construct a multi-omics and immunological profile of systemic inflammation and non-AIDS comorbidities (Fig. 1a), we conducted genome-wide genomics (Illumina, GSA), epigenomics (Illumina, EpicArray), transcriptomics (bulk RNA sequencing), targeted proteomics (Olink Explore 3072) and metabolomics (General Metabolicsʼ untargeted platform), and we assessed ex vivo cytokine production capacity of peripheral blood mononuclear cells (PBMCs) upon simulations with inactivated pathogens. The stimulants used included poly I:C, lipopolysaccharide (LPS), imiquimod, interleukin (IL)-1a, HIV viral envelope, cytomegalovirus (CMV), *Streptococcus pneumoniae*, *Escherichia coli, Staphylococcus aureus*, *Mycobacterium tuberculosis*, *Candida albicans* (conidia), *Candida albicans* (hyphae) and phytohaemagglutinin (PHA) (Methods). To inspect the clinically relevant correlates of molecular variation, we identified LFs, resulting from the combination of multiple omics layers, and associated them with a range of common non-AIDS comorbidities (Fig. 1b). Furthermore, we disentangled the genetic bases of the inter-individual variation of the various omics layers (Fig. 1c). Lastly, we deciphered the causal determinants of immune responses in PLHIV (Fig. 1d).

### Multi-omics LFs capture non-AIDS comorbidities

**Fig. 2:**
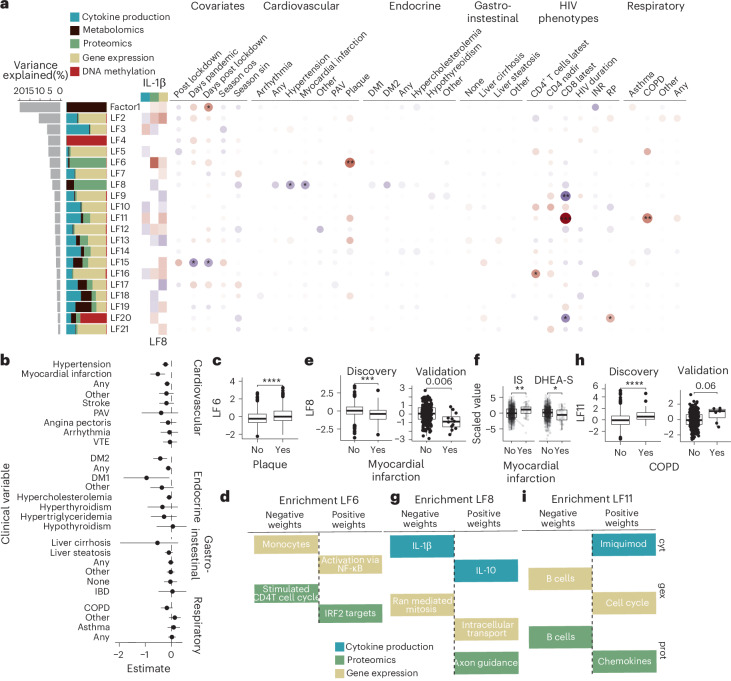
Multi-omic LFs underlying clinical variables in PLHIV. **a**, Left, variance explained by each of the 21 LFs, in percentage. Colored bar plot indicates the proportion of the percentage of variance explained in each of the data modalities. Heatmap indicates the correlation between each of the LFs and IL-1β expression in cytokine production upon stimulation, plasma protein concentration and gene expression. Only significant correlations after FDR correction are colored. Right, dot plot, association of LFs with covariates and clinical variables. Wilcoxon rank-sum test was used for binary variables, Kruskal–Wallis for categorical variables and Pearson’s correlation for continuous variables. Color indicates the direction of the association times its significance (−log_10_ FDR), and size indicates the significance of the association. *FDR < 0.05, **FDR < 0.01, ***FDR < 0.001. **b**, Effect estimates, derived by linear modeling, of increased LF8 values and different clinical variables, including cardiovascular, endocrine, gastrointestinal and respiratory endpoints. Error bars correspond to the limits of the 95% conficence interval. **c**,**e**,**h**, Association of the multi-omic factor values with clinical parameters. *P* values: 2.54 × 10^−6^ (**c**), 2.96 × 10^−4^ (**e**) and 1.69 × 10^−5^ (**h**). **f**, Association of two LF8-related metabolites, indoxyl sulphate (IS) (*P* = 0.003) and DHEA-S (*P* = 0.044), and myocardial infarction. **d**,**g**,**i**, Enrichment in features with positive and negative weights for each factor. *x* axis indicates the direction of enrichment (MOFA pathway enrichment). **c**,**e**,**f**,**h**, Two-sided Wilcoxon rank-sum test, *P < 0.05, **P < 0.01, ***P < 0.001. *n*_discovery_ = 1,075, *n*_validation_ = 267. Box plots show the median (center), first and third quartiles (bounds) and 1.5 times the interquantile range (whiskers). cos, cosine; cyt, cytokine; gex, gene expression; IBD, inflammatory bowel disease; INR, immunological non-responder; PAV, peripheral arterial vascular disease; prot, proteomics; RP, rapid progressor; sin, sine; VTE, venous thromboembolism.

We conducted MOFA^14^ to identify factors that capture inter-individual variation at all data modalities except genomics. This analysis yielded 21 LFs explaining at least 1% of the total variance in the data (1–20% of variance explained; Fig. 2a). Of note, these LFs predominantly comprised molecules sourced from all five data modalities, indicating inter-individual variation in biological processes captured across omics layers through this integration.

We tested the association of each LF with inflammation and clinical variables to gain insights into the biological processes captured by the LFs (Fig. 2a). We observed that most of the factors (19/21 LFs) were significantly correlated (Pearson’s correlation, false discovery rate (FDR) < 0.05; Supplementary Data 1) with systemic inflammation, as measured by IL-1β production capacity upon stimulation, IL-1β plasma concentration or *IL1B* gene expression in PBMCs. This suggests a major role of IL-1β-driven systemic inflammation in the inter-individual variability of PLHIV. LFs were associated (Supplementary Data 2) with comorbidities and HIV phenotypes. Thus, LFs not only capture inter-individual variation in inflammation but also link to non-AIDS comorbidities.

Next, we tested the association between LFs and HIV-related phenotypes (Supplementary Data 2). We observed five significant associations (FDR < 0.05; Supplementary Data 2) with three HIV-related phenotypes (rapid progressors (individuals with a sharp decrease of CD4^+^ T cell counts after infection^15^) and CD8^+^ and CD4^+^ T cell counts). This indicates that the inter-individual variation at molecular levels in our cohort was driven more by non-HIV-related phenotypes rather than the HIV-related phenotypes tested. A set of LFs was found to elucidate the molecular pathways underlying comorbidities: the presence of a carotid plaque (LF6), documented CVD (hypertension and/or myocardial infarction) and endocrine disorders (LF8) and chronic obstructive pulmonary disease (COPD) (LF11) in PLHIV (Fig. 2).

#### A proteomic profile of immune activation higher in patients with a carotid plaque

**Extended Data Fig. 1:**
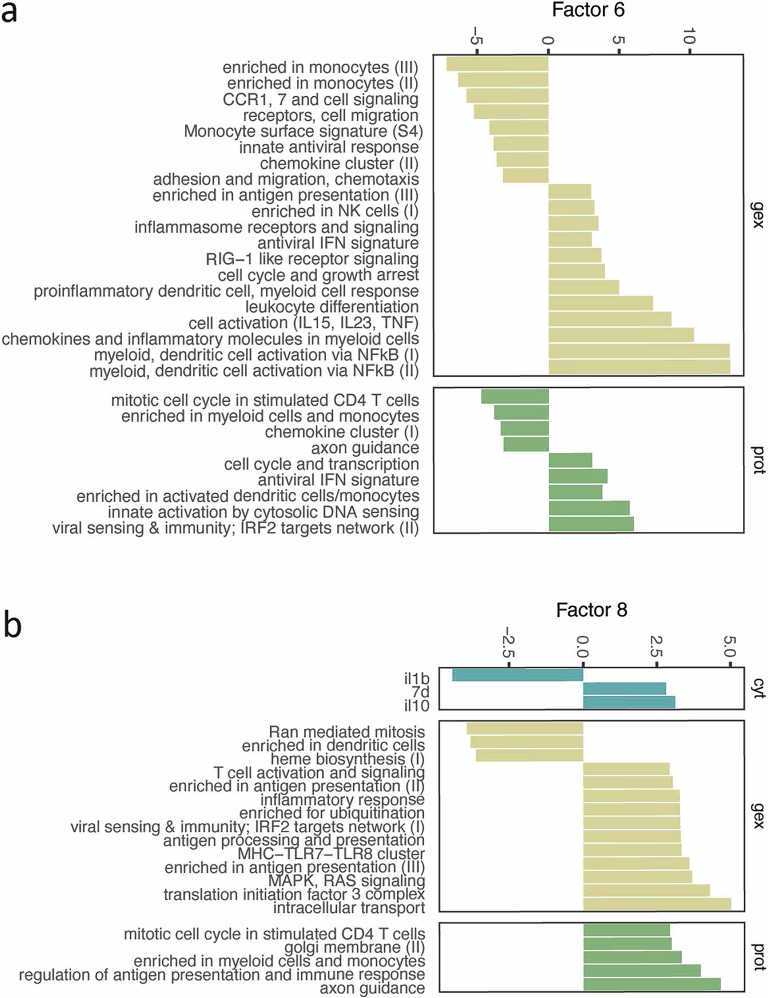
Enrichment profiles. **a**, Enrichment profile for latent factor 6. **b**, Enrichment profile of latent factor 8.

We identified a multi-omic factor, LF6, which mainly comprised plasma protein concentrations and which was significantly higher in PLHIV in whom a carotid plaque was documented (Fig. 2c). LF6 explained 5.19% of the variation across layers and captured differences in innate immune activation and NF-κB activation at the proteomic and gene expression level (Fig. 2d and Extended Data Fig. 1a).

#### A protein and metabolite profile linked to lower cardiovascular and endocrine comorbidities in PLHIV

The presence of CVDs or endocrine disorders was associated with LF8, a factor accounting for 3.89% of the total variance in the omics data and mainly consisting of molecules measured at metabolomics and proteomics levels (Fig. 2a,b). Specifically, LF8 was negatively associated with myocardial infarction (Fig. 2e) and hypertension (Fig. 2a) (FDR < 0.05) and had a consistent negative association with other cardiovascular and endocrine diseases that were documented in the cohort (Fig. 2b). A part of the metabolites and proteins constructing LF8 was significantly different between PLHIV with myocardial infarction or other cardiovascular disorders, including indoxyl sulphate and dehydroepiandrosterone sulphate (DHEA-S) (Fig. 2f). Indoxyl sulphate is a gut-derived metabolite that impairs CD4 function in HIV^16^ and is a risk factor for CVD^17^. DHEA-S is a circulating steroid hormone metabolized from DHEA^18^, which has been studied for its modulatory properties in HIV infection^19^. Both metabolites contribute to an overall set of metabolites and proteins that could serve as biomarkers for metabolic comorbidities in PLHIV (Supplementary Data 6–26). Furthermore, LF8 captured the variation in IL-10 and IL-1β cytokine responses to stimulation as well as RNA-mediated mitosis and intracellular transport (Fig. 2g and Extended Data Fig. 1b).

#### CD8^+^ T cell function is linked with COPD in PLHIV

**Extended Data Fig. 2:**
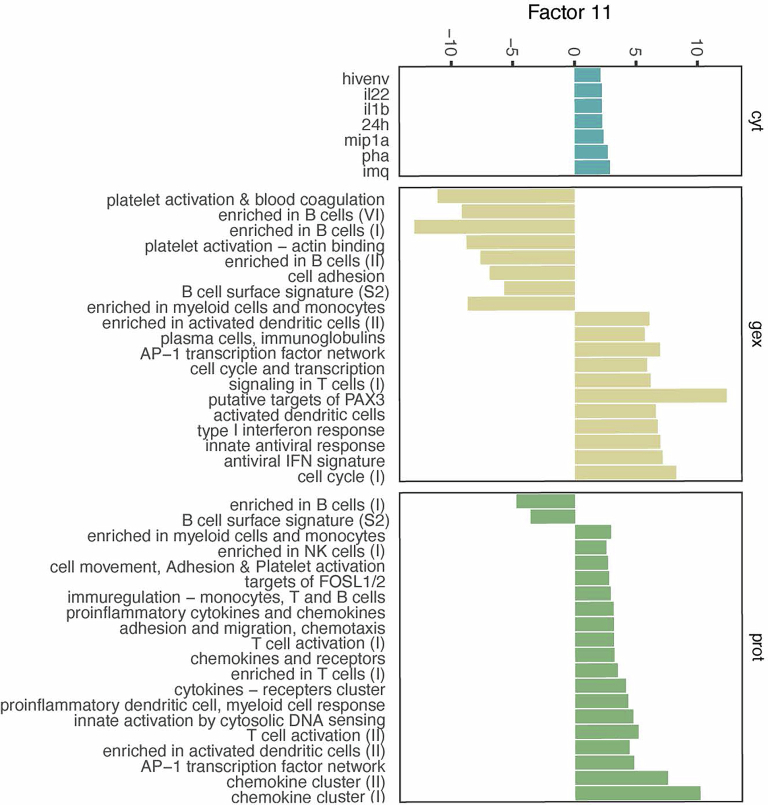
**Enrichment profile for latent factor 11**.

COPD was positively associated with LF11, which was significantly correlated with CD8^+^ T cell counts (Fig. 2a,h). LF11 was associated with lower B-cell-related proteins and transcripts, together with higher interferon (IFN) activity and chemokine and T cell function (Fig. 2i and Extended Data Fig. 2).

#### A multi-omic profile linked to rapid progressors

**Extended Data Fig. 3:**
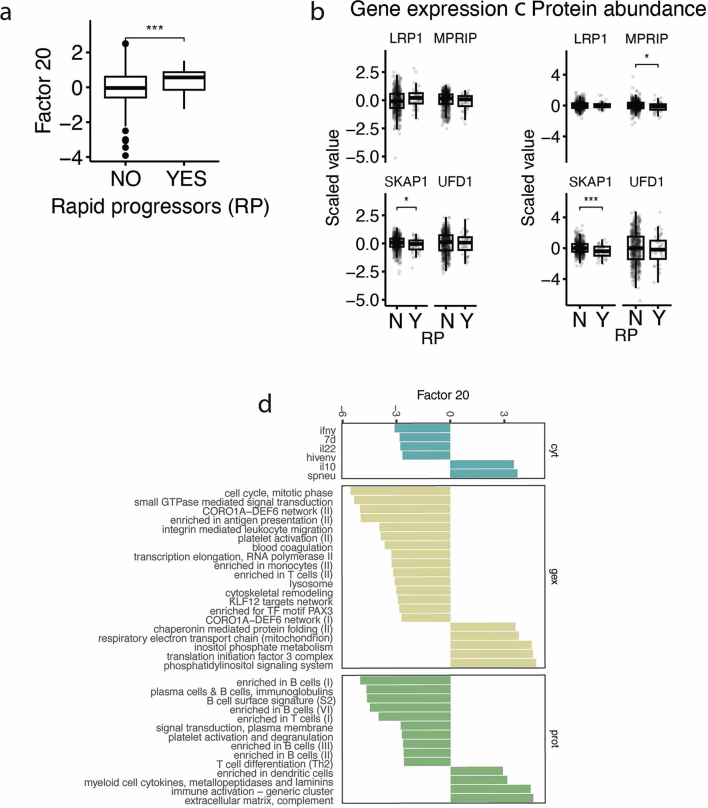
Overview of latent factor 20. **a**, Association of latent factor 20 with rapid progression. **b**,**c**, Features with significant weights in gene expression, examined in gene expression and protein abundances. Wilcoxon rank-sum test *P < 0.05 **P < 0.01 ***P < 0.001 ****P < 0.0001. **d**, Enrichment profile for latent factor 20.

LF20, a factor dominated by the whole blood DNA methylation layer (Extended Data Fig. 3a), was significantly associated with rapid progressors: individuals with a sharp decrease of CD4^+^ T cell counts after infection^15^ (Fig. 2a). To understand the biological function underlying LF20, we examined the gene expression and protein abundance of genes with a significant weight contributing to LF20 in rapid progressors compared to the rest. This highlighted *SKAP1* as a marker with lower expression in rapid progressors, both in gene expression in PBMCs and protein abundance in plasma (Extended Data Fig. 3b,c). We observed a consistent pathway enrichment of inositol metabolism at the gene expression level and immune activation at the protein level (Extended Data Fig. 3d). This suggests a common multi-omics profile present in individuals with a previous history of rapid progression during HIV infection.

### The genetic regulation of molecular traits in PLHIV

**Fig. 3:**
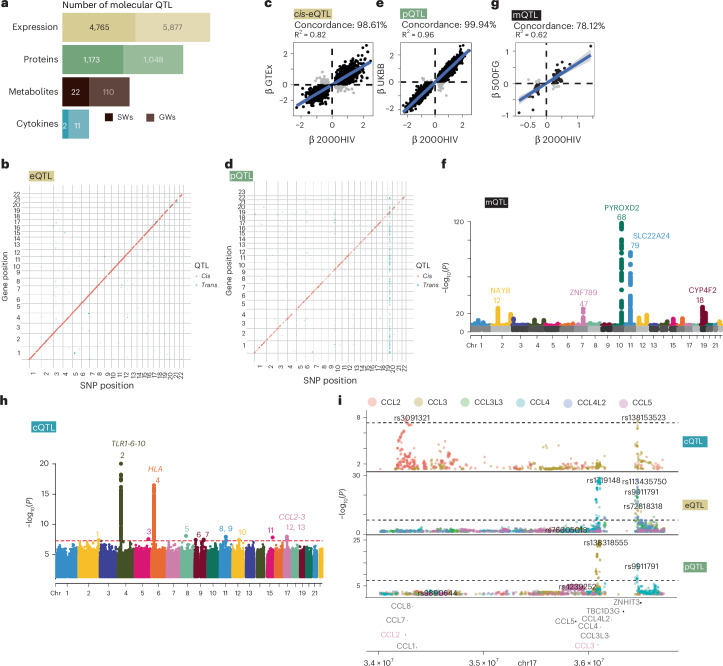
Multi-omics genetic regulation of PLHIV. **a**, Number of loci identified per molecular layer. **b**,**d**,**f**,**h**, SWs QTL; only significant associations are shown. SWs is defined by correcting the GWs threshold (*P* < 5 × 10^−8^) by the number of effective tests (Methods). **b**, eQTL. **d**, pQTL. **f**, mQTL. **h**, cQTL. **c**,**e**,**g**, Comparison of effects between QTL in PLHIV and healthy individuals. Gray shaded area indicates the 95% confidence interval. **c**, Compared to eQTL in whole blood in GTEx. **d**, Compared to pQTL in plasma in the UK Biobank. **e**, Compared to metabolite QTL in the 500 Functional Genomics Project (500FG). **i**, LocusZoom plot of the CCL2–CCL3 hotspot, two contiguous loci harboring regulation of immune responses, plasma proteins and gene expression. Dotted line indicates the GWs threshold (*P* < 5 × 10^−8^). 7d, 7 days; calbhy, *C. albicans* (hyphae); cmv, cytomegalovirus; ecoli, *E. coli*; HIVenv, HIV viral envelope; il1a, IL-1A; imq, imiquimod; lps, lipopolysaccharide; mtb, *Mycobacterium tuberculosis*; pha, polyhydroxyalkanoate; polyic, poly I:C; saureus, *S. aureus;* spneu, *S. pneumoniae*.

**Extended Data Fig. 4:**
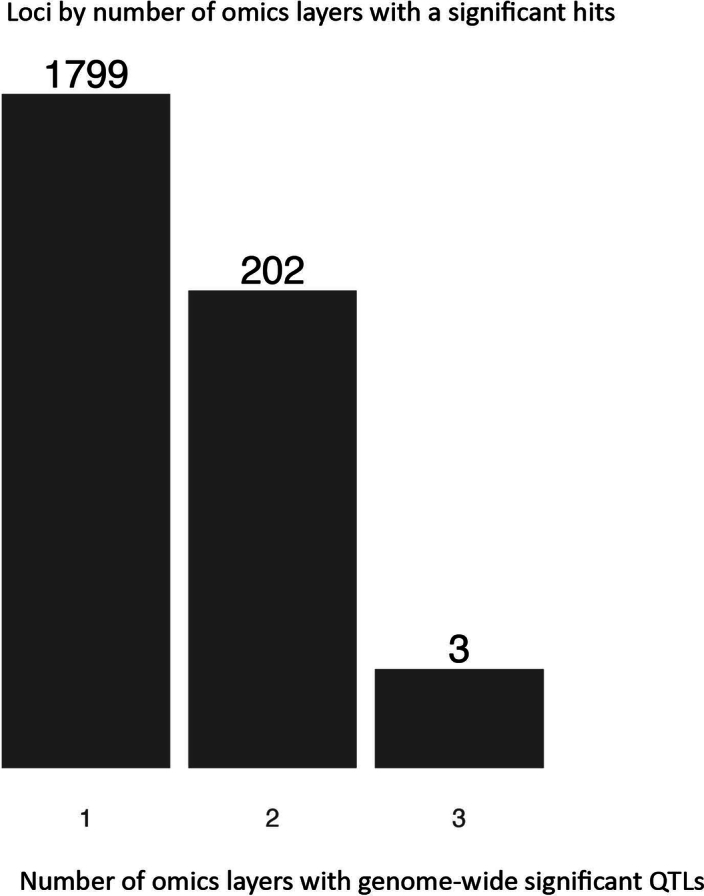
Genetic loci by number of layers in which at least one genome-wide significant hit was found. Results show per locus the SNP with the highest number of molecular layers with a significant QTL.

To understand the role of genetic variation in determining the molecular phenotypes of PLHIV, we performed QTL mapping on multi-omics and immune profiles in both the discovery and validation cohorts (Fig. 1c). We defined two levels of significant QTL: genome-wide significance (GWs) for associations with a *P* value below the GWs threshold (*P* < 5 × 10^−8^) and study-wide significance (SWs) by applying multiple-testing correction on the GWs threshold considering the number of independent features tested (Methods). In total, we identified 5,962 molecular QTL (SWs), finding the highest number in gene expression, followed by proteomics, metabolomics and immune function (Fig. 3a). Notably, three loci harboring missense variants—*NLRP12*, *TLR1* and *KLKB1*—were shown to regulate three of the layers described below (Extended Data Fig. 4).

**Extended Data Fig. 5:**
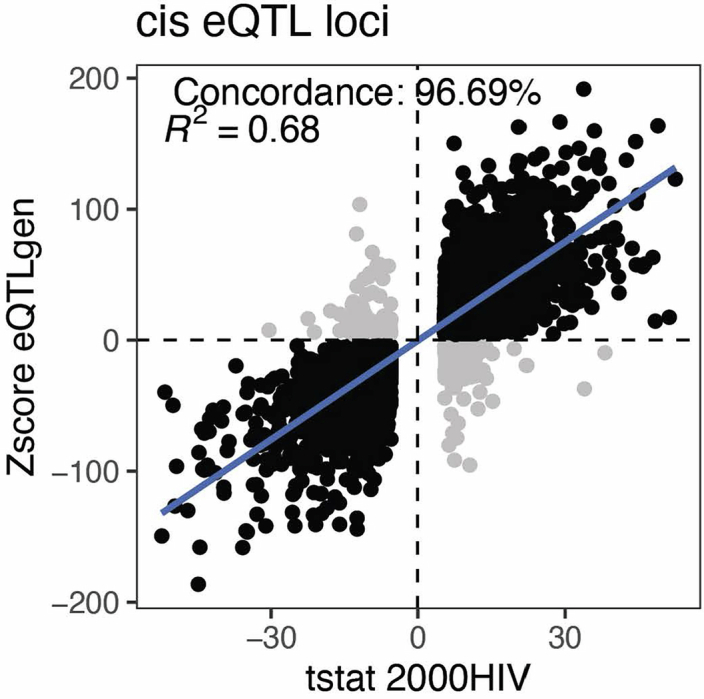
Comparison of effects between cis eQTL in PLHIV and healthy individuals from eQTLgen. Top SNPs for each genome-wide significant locus were tested in the eQTLgen consortium.

We performed expression QTL (eQTL) mapping for 17,741 genes to identify genetic variants regulating gene expression levels in PBMCs of PLHIV (Fig. 3b). Our analysis revealed 10,642 GWs and 4,765 SWs eQTL for 8,591 and 4,920 genes, respectively. We compared the *cis*-eQTL found in PLHIV with those from two different datasets, eQTLGen^20^ and Genotype-Tissue Expression (GTEx)^21^, and observed concordance in the direction of effects (98.6% for GTEx, 94.9% for eQTLGen) and high correlation (R^2^ = 0.82 for GTEx, R^2^ = 0.68 for eQTLGen; Fig. 3c and Extended Data Fig. 5). These findings indicate that *cis* genetic regulation of gene expression does not differ between PLHIV and healthy individuals. Interestingly, we identified 42 significant *cis*-eQTL that had a discordant effect in PLHIV compared to healthy individuals in both GTEx and eQTLGen (Supplementary Data 4), suggesting disease-specific genetic regulation.

Our QTL analysis of 2,367 plasma proteins in PLHIV identified 3,019 GWs protein QTL (pQTL) for 1,427 proteins and 1,646 SWs pQTL for 1,040 proteins, which covered 43.93% of all measured proteins (Fig. 3d). A hotspot on chromosome 19 could be highlighted, showing *trans* signals for 367 proteins. A comparative analysis of pQTL effect estimates between PLHIV and a population-based cohort from the UK Biobank Pharma Proteomics Project (UKB-PPP) initiative^22^ revealed a concordance of direction in 99.94% of the GWs pQTL effects tested, indicating a common regulation of protein abundances between PLHIV and healthy people (Fig. 3e).

Mapping metabolite abundances in plasma to genetic variation, we found 171 GWs metabolite QTL (mQTL) for 159 metabolites and 40 SWs mQTL for 1,040 endogenous metabolites (Fig. 3f). Comparing our results to those from a healthy cohort^23^, we found high concordance in the effects by the sentinel single-nucleotide polymorphisms (SNPs) of the GWs loci (R^2^ = 0.62; Fig. 3g). The lower concordance observed with genetic variance linked to circulating metabolites, compared to those seen with protein and gene expression, may be attributed to the effects of HIV or ART on metabolic processes^24^.

Lastly, we performed cytokine QTL (cQTL) mapping and revealed two SWs and 13 GWs cQTL, of which 12 were not previously reported^25–27^ (Fig. 3h). The cQTL highlighted two known polymorphic regions for immune response: the *TLR1-6-10* locus^27^ and the *HLA* locus. Interestingly, we identified two *cis*-acting loci regulating CCL2 (MCP-1) and CCL3 (MIP-1ɑ) at the cytokine, gene expression and protein concentration levels (Fig. 3i). We found co-localizations between the CCL3 response locus and the baseline *CCL3* eQTL (H4 = 0.861), as well as neighboring genes *CCL3L3* (H4 = 0.995) and *CCL4L2* (H4 = 0.996) and protein CCL4 (H4 = 0.994). By contrast, no SNPs were associated with baseline *CCL2* protein abundance within the CCL2 response locus. Additionally, for pQTL in healthy individuals^22^, the lead SNP (rs3091321) was not significantly associated with CCL2 protein abundance (*P* = 0.28) nor in GTEx’s whole blood eQTL^21^. This indicates that the SNPs influencing CCL3 response are also influencing CCL3 at baseline, whereas the CCL2 response genetic regulation is specific to settings with immune stimulation.

Overall, this study provided a comprehensive genetic map detailing gene expression, protein abundance, metabolite abundance and cytokine production in PLHIV. Our findings reveal a largely consistent genetic regulatory pattern compared to healthy individuals as well as genetic regulators influencing CCL2 and CCL3 at various omics layers. This study provides a comprehensive repository of genetic effects across different molecular layers in PLHIV.

### Causality between circulating molecules and immune function

**Fig. 4:**
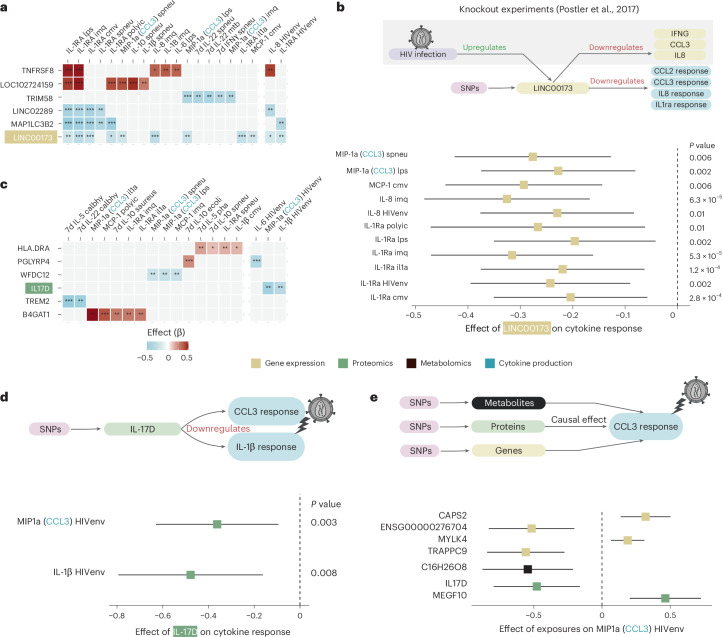
The causal link between circulating markers and the immune response of PLHIV. **a**,**c**, Top genes (**a**) and proteins (**c**) with the most significant Mendelian randomization (MR) links to cytokine responses. Significance was estimated by IVW MR with sensitivity checks (Methods). Color indicates the effect calculated by IVW MR. *P < 0.05, **P < 0.01, ***P < 0.001. **b**,**d**,**e**, Examples of regulators of immune response in PLHIV. All effects and *P* values were calculated by IVW MR. Only significant effects (*P* < 0.05 and sensitivity checks passed) are shown. **b**, All cytokine responses regulated by *LINC00173*. **d**, Responses to the HIV envelope regulated by IL-17D. **e**, All the regulators of CCL3 responses to the HIV envelope. Schematics were created with BioRender.com.

Immune dysfunction increases the risk of developing non-AIDS comorbidities in PLHIV. To investigate how circulating molecules causally regulate the immune response in PLHIV, we performed Mendelian randomization including clumped SNPs significantly associated with the exposure (*P*_discovery_ < 1 × 10^−5^ and *P*_validation_ < 0.05; Methods). We identified 313 genes, 55 proteins and 14 metabolites that show significant causal association with cytokine responses upon stimulation in PLHIV. Notably, six genes were causally linked with at least six different cytokine stimulation pairs (Fig. 4a), with *LINC00173* showing the highest number of causal links with 11 different cytokine stimulation pairs. Specifically, higher *LINC00173* expression was associated with lower cytokine responses, particularly with IL-1Ra production (Fig. 4b). This underscores the potential regulatory role of this long non-coding RNA (lncRNA) in the immune responses of PLHIV, consistent with previous findings where *LINC00173* was found to be upregulated during HIV infection and regulating multiple cytokine responses^28^.

We identified six circulating plasma proteins that were causally associated with at least three cytokine stimulation pairs (Fig. 4c). Interestingly, IL-17D was causally related to the production of two cytokines upon HIV envelope stimulation, in which higher IL-17D concentration was linked with a lower CCL3 and IL-1β production (Fig. 4d), suggesting a modulatory potential for reducing persistent immune activation in PLHIV. Based on Mendelian randomization analysis, two genes (*RP11-128N14.4*, a lncRNA, and *TRAPPC9*), a metabolite and a protein (IL-17D, described above) causally downregulate CCL3 upon HIV envelope stimulation, whereas two genes (*CAPS2* and *MYLK4*) and a protein (MEGF10) upregulate CCL3 upon HIV envelope stimulation (Fig. 4e). In summary, we identified multi-omic causal factors of immune responses in PLHIV.

### The *NLRP12* locus regulates the inflammasome levels in PLHIV

**Fig. 5:**
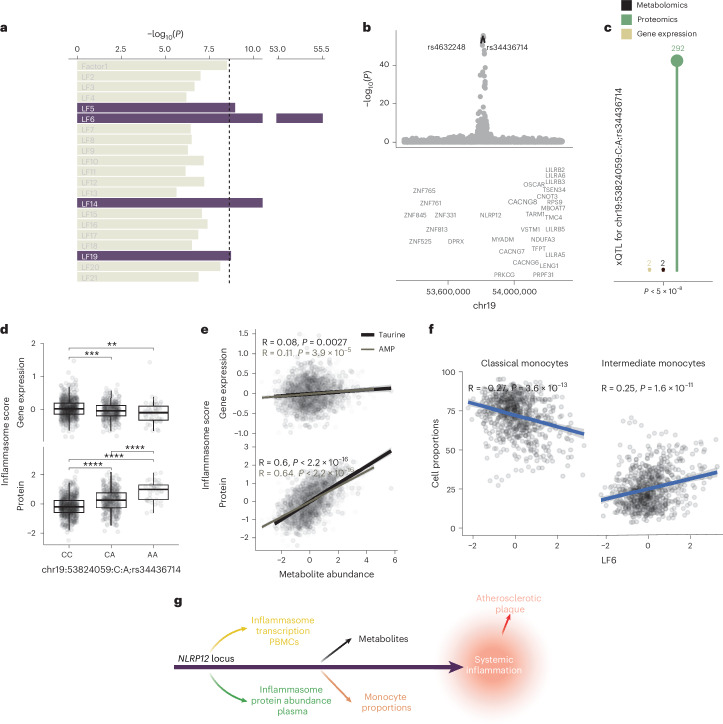
The *NLRP12* locus and the multi-omics landscape of the inflammasome in PLHIV. **a**, Top association between LFs and genome-wide variants by QTL mapping. **b**, LocusZoom plot of the association between the *NLRP12* locus and LF6. **c**, Number of GWs (*P* < 5 × 10^−8^) associations replicated in the validation cohort (*P* < 0.05) between the missense variant rs34436714 and different omics layers. **d**, Association between rs34436714 and an inflammasome score computed at gene expression and protein levels. Discovery cohort, *n* = 1,075. Two-sided pairwise Wilcoxon rank-sum test, *P < 0.05, **P < 0.01, ***P < 0.01. Box plots show the median (center), first and third quartiles (bounds) and 1.5 times the interquantile range (whiskers). **e**, Correlation between AMP and taurine abundance and the inflammasome score computed at gene expression and protein levels. Pearson’s correlation. **f**, Correlation between LF6 and two monocyte proportions. Pearson’s correlation. The gray shaded area indicates the 95% confidence interval. **g**, Schematic of the hypothesized mechanism. Schematics were created with BioRender.com.

**Extended Data Fig. 6:**
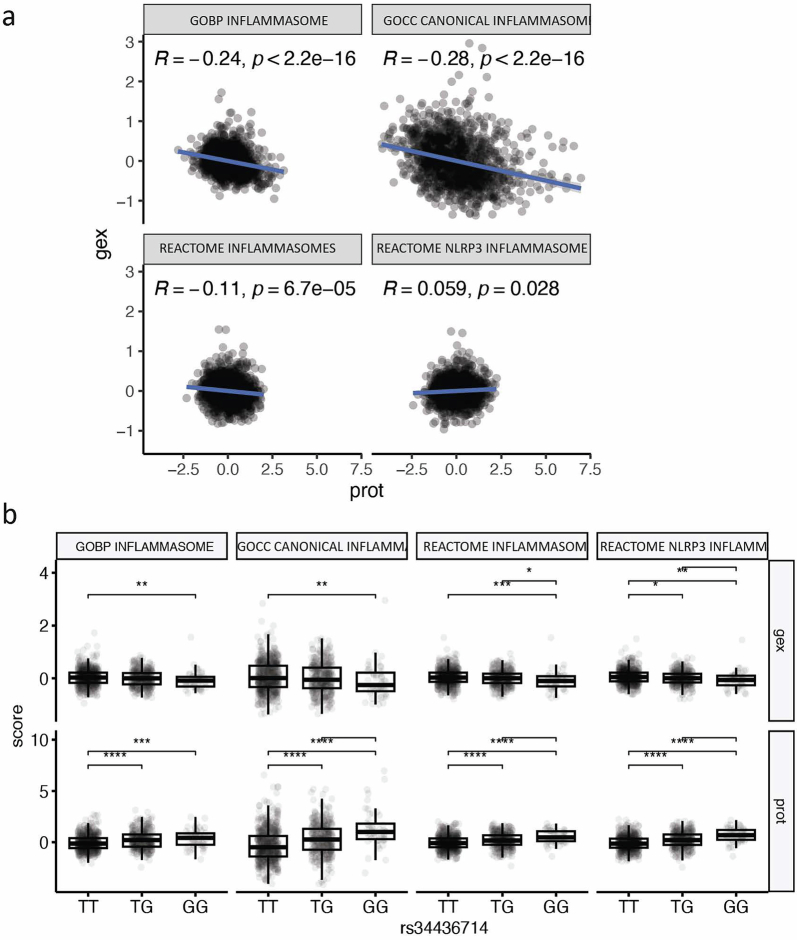
Inflammasome scores in gene expression in PBMCs and protein abundance in Plasma. **a**, Spearman correlation for scores between gene expression and protein abundance. Calculated based on four different gene sets: Gene Ontology (GO) biological processes (BP), GO cellular component (CC) and Reactome. **b**, Association of rs34436714 with the four different gene sets.

**Extended Data Fig. 7:**
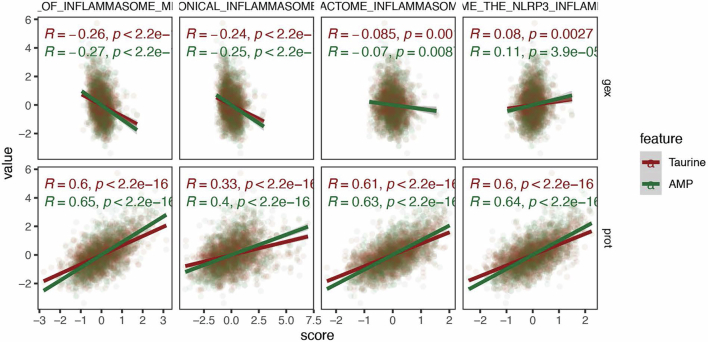
Correlation between the inflammasome score in gene expression in PBMCs and protein abundance in plasma and plasma metabolite abundances of AMP and Taurine. Spearman correlation.

We investigated the genetic determinants underlying the inter-individual variation of identified LFs linked with systemic inflammation through QTL mapping. This analysis revealed four SWs loci associated with four LFs (Fig. 5a and Supplementary Data 5). The strongest association was observed between LF6, an LF related to inflammasome factors (Supplementary Data 11), and the *NLRP12* locus, a pQTL hotspot regulating multiple proteins (Fig. 3d). Although *NLRP12* was implied as an artifact in pQTL studies in individuals without HIV^22^, our findings demonstrate its pleiotropic effects. The missense variant rs34436714 was linked to the lead variant (R^2^ > 0.99) and showed significant associations with two genes, two metabolites and 292 proteins (Fig. 5c). Furthermore, the G allele of rs34436714 was positively associated with the concentration of NLRP3 inflammasome proteins and negatively associated with the transcription of its genes (Fig. 5d), despite the existing correlation between protein and gene expression levels (Extended Data Fig. 6). Metabolites associated with the *NLRP12* locus, namely adenosine monophosphate (AMP) and taurine, also showed positive associations with NLRP3 inflammasome factors at both gene expression and protein levels (Fig. 5e and Extended Data Fig. 7). Furthermore, measuring immune cell proportions showed that LF6, the inflammasome factor regulated by NLRP12, correlated with monocyte subpopulations (Fig. 5f), underscoring the pleiotropic effects of the *NLRP12* locus in regulating inflammation.

Thus, the *NLRP12* locus systemically regulates the inflammasome pathway, which drives systemic inflammation in PLHIV and contributes to carotid plaque formation, at different molecular layers (Fig. 5g).

## Discussion

In this study, we characterized the molecular and genetic determinants of inter-individual variation of virally suppressed PLHIV. We integrated five omics layers and immune function to identify multi-omics LFs that captured processes underlying comorbidities. We found LFs associated with CVD, COPD, rapid progressors and carotid plaque. We mapped genetic variation to four distinct omics layers to dissect the genetic determinants of molecular diversity in PLHIV, showing a broadly similar genetic regulation when compared to individuals without HIV. We inspected the causal link between circulating molecules and immune response, providing a unique resource and insights into potential immune modulators in PLHIV. Lastly, we uncovered the inflammasome as a key factor driving systemic inflammation and comorbidities in PLHIV and genetically regulated at multiple omics layers by the *NLRP12* locus.

Immunomodulators have emerged as a possible therapeutic strategy to tackle systemic inflammation and, therefore, comorbidities in virally suppressed PLHIV^29–31^. Some of these therapies specifically target cytokines, which are known drivers of inflammation. We subsequently assessed the influence of common genetic variants on cytokine production. The cQTL were concordant with those found in the general population^25^ and provided two approaches that may aid in immunomodulation. The first approach is targeting genes and proteins pinpointed by loci that regulate the immune response in PLHIV^32^. Another approach is using genetic factors to stratify those individuals at higher risk of developing an exacerbated inflammatory response^33^. Moreover, cQTL revealed two relevant loci for HIV pathophysiology, *CCL2* and *CCL3*. CCL2, with its immune cell recruiting properties, has been linked to early seeding of the latent HIV reservoir^34^ and HIV-associated comorbidities, such as HIV-related dementia^35^. CCL3 is a ligand for CCR5, the entry co-receptor for the HIV virus^36^; CCR2b, the receptor for CCL2, is also a co-receptor for HIV^37^.

Building on the genetic landscape outlined by the QTL analysis, we employed the associations found for gene expression, proteins and metabolites to establish causal links to cytokine production using Mendelian randomization. We causally linked *LINC00173*, a lncRNA, to a total of 11 cytokine stimulations (across four unique cytokines) in PLHIV. *LINC00173* is downregulated upon T cell activation through PMA and ionomycin stimulation, leading to the upregulation of cytokines such as IL-8, CCL3 and IFNγ, whereas it is upregulated upon HIV infection, suggesting that HIV may leverage this lncRNA to module immune functions^28^. Our Mendelian randomization analysis reveals the substantial influence of *LINC00173* on different cytokines, namely IL-8, CCL3, CCL2 and IL-1Ra, upon various stimulations, including the HIV envelope. Furthermore, we found six significant causal links between higher expression levels of *LINC00173* and lower IL-1Ra production. IL-1Ra is the natural antagonist of the IL-1 receptor and, in its recombinant form (anakinra), is used as an immunomodulatory drug in multiple infections^38^. This indicates a potential regulatory role of *LINC00173* in immune activation through modulation of IL-1 signaling in PLHIV.

The NLRP3 inflammasome is a key component of atherosclerosis in PLHIV and the general population^39^. In our study, we observed a higher abundance of inflammasome proteins in individuals with carotid plaque. Interestingly, we found that the *NLRP12* locus regulates the inflammasome. *NLRP12* was previously found in protein levels in healthy individuals but was overlooked as a potential artifact^22^. A missense variant in the locus, rs34436714, was associated with the inflammasome in multiple molecular layers. rs34436714 was associated with the NLRP3 inflammasome at the transcriptomic, proteomic and metabolomic levels. This provides compelling evidence for the key role of *NLRP12* and its variants in regulating systemic levels of the inflammasome. NLRP12 impedes the activation of the NLRP3 inflammasome^40^ and has been shown to attenuate inflammation in the joints by modulating Th17 activation^41^ and in the colon by regulating the innate response to gut dysbiosis^42,43^, one of the roots of systemic inflammation in PLHIV. Thus, further studies on the role of *NLRP12* and its missense variant rs34436714 in PLHIV may provide more information on the causes and possible therapies of systemic inflammation.

A limitation of the analyses presented here is the translatability of our findings to different genetic ancestries and to both biological sexes. Although the 2000HIV study recruited 15.2% female participants and 17.5% participants of non-European genetic ancestry^14^, our analyses here focused on the determinants of inter-individual variation in people of European ancestry without considering sex-specific effects. A more tailored analysis, highlighting the sex-specific associations and genetic regulations, may aid in a better understanding of the sex differences in non-AIDS-related comorbidities and inflammation. Furthermore, adding the other genetic ancestries in the study can help to identify ancestry-specific effects on immune function and comorbidities.

In summary, our study provides a comprehensive multi-omics investigation of PLHIV, inspecting their genetic regulation, the determinants of their immune function and the mechanisms underlying comorbidities. We identified 21 LFs encompassing 41,036 features across five data modalities, and we pinpointed 17,595 loci that regulate four data layers and 386 molecules from three data modalities that causally modulate immune responses in PLHIV. All these results are accessible through our web tool, as a resource of inter-individual variation in PLHIV, at https://lab-li.ciim-hannover.de/apps/hiv_xqtl_atlas.

## Methods

### Participants from the 2000HIV study

The 2000HIV study is a prospective multicentric observational longitudinal cohort of virally suppressed PLHIV^15^. Participants were recruited from October 2019 to October 2021. The cohort included both discovery and validation cohorts. Participants in the discovery cohort were recruited from three specialized Dutch HIV treatment facilities, two university medical centers and one large general hospital (Radboudumc Nijmegen, Erasmus MC Rotterdam and OLVG Amsterdam). Participants in the validation cohort were recruited at a different medical facility: a large general hospital (Elisabeth-TweeSteden Ziekenhuis Tilburg). Inclusion criteria included HIV-1 infection, age 18 years or older, 6 months of ART and a most recent HIV-1 RNA level of less than 200 copies per milliliter. Individuals with spontaneous HIV-1 control without ART could participate if viral loads were less than 10,000 copies per milliliter for at least 5 years and CD4^+^ T cell counts were stable (>500 cells per mm^3^). Exclusion criteria included no informed consent, insufficient communication due to language barriers or other issues, current pregnancy, detectable viral hepatitis B or C DNA by polymerase chain reaction or signs of any current acute infection. Extreme clinical phenotypes, such as spontaneous ‘elite’ controllers, and rapid progressors were identified as described previously^15^ as well as carotid plaque assessment using B mode ultrasound.

The 2000HIV study was approved by Independent Review Board Nijmegen (NL68056.091.81) and published at ClinicalTrials.gov (NCT03994835). Written informed consent was received from participants before inclusion in the study. All experiments with human samples were conducted following the principles of the Declaration of Helsinki.

### Multi-omics measurements

#### Genomics—genotyping array

DNA was extracted from each participant’s whole blood. The Illumina Infinium Global Screening Array was used for genotyping all participants of multiple ethnicities in the 2000HIV cohort. PLINK version 1.90b^44^ was used to perform quality control on raw variants and samples before imputation. The dataset excluded genetic variants with call rate genotype missingness of more than 5% and deviations from Hardy–Weinberg equilibrium (HWE) with *P* < 10^−6^. The HWE exact test was performed with variants stratified by ethnicity. We excluded samples with a call rate of less than 97.5% and heterozygosity rates that deviated more than 3 s.d. from the mean rate per self-reported ethnicity. Genetic variants that passed quality control were converted from GRCh37 to GRCh38 using the UCSC liftOver tool^45^. Next, TOPMed Freeze 5 was used on genome build GRCh38 to align strands to the TOPMed reference panel. We used the McCarthy group tools for alignment (https://www.well.ox.ac.uk/~wrayner/tools/). After quality control, 582,404 variants from 1,864 individuals were kept for the imputation process. The filtered raw variants were uploaded to the TOPMed Imputation server and compared to the TOPMed (version r2 on GRCh38) reference panel. The imputed variants were filtered using BCFtools stratified by ethnicity^46^, excluding variants with low imputation quality scores (*R*^2^ < 0.3 or empirical *R*^2^ < 0.7) or minor allele frequency (MAF) < 1%. This yielded 10,810,841 variants from 1,864 members of the 2000HIV multi-ancestry cohort.

The above-mentioned quality control and imputation procedure was applied independently to the European ancestry discovery and validation cohorts. During quality control per marker, variants with a call rate greater than 5%, MAF < 1% and deviation from HWE (*P* < 10^−6^) were removed from the European datasets of the discovery and validation cohorts. Samples with a call rate less than 97.5%, heterozygosity rates that deviated over 3 s.d. from the mean and ethnic outliers identified through principal component analysis (PCA) were removed during quality control. Individuals were defined as ethnic outliers if their genetic principal component 1 (PC1) and/or principal component 2 (PC2) deviated by more than 3 s.d. from the mean PC1 and/or PC2 of the European population from the 1000 Genomes Project^47^. After quality control and imputation, the imputed variants were filtered as described above, yielding 9,148,674 and 9,130,602 SNPs from 1,003 and 257 individuals in the discovery and validation cohorts, respectively.

After imputation, PLINK 2.0 (ref. ^48^) was used to perform quality control. Any variants failing the HWE test at *P* < 1 × 10^−12^ and those with MAF < 1% and *R*^2^ < 0.05 were eliminated. In total, 8,944,122 imputed SNP variants were kept for further analysis after quality control.

#### Epigenomics—methylation array

A total of 1,914 samples underwent DNA methylation. The Radboudumc Genetics Department isolated DNA from EDTA whole blood using the chemagic STAR automated configuration (consisting of the Microlab STAR and Chemagen Magnetic Separation Module 1; Hamilton Robotics) combined with Chemagen nucleic acid extraction technology with magnetic polyvinyl alcohol (M-PVA) beads, which follows a standard and automated bind–wash–elute protocol. A NanoDrop spectrophotometer was used to determine the DNA concentration and 260/280-nm ratio. Samples were then normalized to 50 ng µl^−1^ in TE buffer and randomly distributed among plates. The Illumina Infinium MethylationEPIC BeadChip array was used to profile DNA methylation across the genome. Standard sample-based and probe-based quality control was carried out. DNA methylation values were estimated from raw IDAT files using R’s ‘minfi’ package (version 4.2.0). Preprocessing steps eliminated two sex mismatch samples from the discovery cohort and one low-quality sample from the validation cohort (call rate <99%). Probes (discovery: *n* = 2,743 and validation: *n* = 2,641) with missing methylation values (detection *P* > 0.01) in more than 10% of samples, as well as probes on the sex chromosome (*n* = 19,627), were excluded from the downstream analysis. Because the participants are European, we also removed probes containing SNPs at target CpG sites with MAF > 5% in European populations as well as probes that mapped to multiple loci (both discovery and validation: *n* = 52,173). Next, we applied stratified quantile normalization. Methylation *β*-values were calculated as a percentage: *β* = *M* / (*M* + *U* + 100), where *M* and *U* represent methylated and unmethylated signal intensities, respectively. The *β*-values were then transformed to *M*-values as log_2_(*β* / (1 − *β*)), and *M*-values were used in all subsequent analyses.

#### Transcriptomics—RNA sequencing

For transcriptomics analysis, PBMCs were sequenced in bulk using short-read sequencing with current Illumina technology (>30 million reads per sample). STAR alignment was used to map the sequencing reads to the most recent version of the human reference genome NCBI build 38. Gene expression was estimated using the HTSeqCount function from DESeq2 with the most recent Ensembl gene annotation. The DESeq2 pipeline is used to process raw counts by applying rlog transformation, normalization and exclusion of low abundant transcripts. Further details on the quality control of the transcriptomics data can be found in the paper describing the cohort^15^.

#### Proteomics—Olink platform

Circulating plasma protein expression was measured using a commercially available multiplex proximity extension assay (PEA) from Olink Proteomics AB in three batches. This study used the entire library (Olink Explore 3072), which included 3,072 targeted proteins organized into eight 384-plex panels focusing on inflammatory, oncological, cardiometabolic and neurological proteins. Protein measurements were delivered as normalized protein expression (NPX) values following a quality control and normalization process developed and provided by Olink. NPX values are derived by subtracting the extension control and the plate values from Cq values. A correction factor is applied to shift the scale, and all values are reported in the log_2_scale. Bridging normalization was used to remove batch effects in each of the eight panels from the Olink Explore 3072 platform, and IL-6, TNF, CXCL8, LMOD1, SCRIB and IDO1 were measured as technical duplicates for quality control purposes. Strong correlations were observed between the technical duplicates among panels, and, therefore, we selected the measurements from the inflammatory panel. Next, we excluded proteins with limit of detection (LOD) ≥ 25 of the samples (*n* = 547 proteins were excluded), resulting in 2,367 proteins for follow-up analysis. Next, during quality control per sample, we performed PCA using the NPX. Outliers were defined as those samples falling above or below 4 s.d. from the mean of PC1 and/or PC2. In total, seven samples were excluded based on PCA, resulting in 1,910 samples analyzed. After this, samples from individuals of European genetic ancestry were selected for this analysis.

#### Metabolomics—mass spectrometry

The abundance of 1,720 circulated metabolites in 1,902 serum samples was determined using General Metabolicsʼ untargeted metabolic platform. Untargeted metabolome profiling was carried out on plasma samples using flow injection electrospray time-of-flight mass spectrometry, as described previously, in collaboration with General Metabolics^49^. Metabolites were identified based on the mass-to-charge ratio (ion *m*/*z*). Prior to analysis, the raw metabolome data were averaged and normalized to remove duplicate peak intensity using a moving median normalization. PCA was then used to identify potential outlier samples. Metabolites were annotated and classified according to the metabolomic source (endogenous, food or drug) and chemical taxonomy using publicly available data from the Human Metabolome Database (HMDB)^50^. According to the HMDB, 851 of the 1,720 metabolites were identified as endogenous and were used for further analysis.

#### Cytokine production assay

PBMCs were stimulated with a range of whole (inactivated) pathogens, pattern recognition receptor ligands, other pathogen-derived antigens and viral stimuli to quantify the capacity for cytokine production. Round-bottom 96-well plates (Greiner Bio-One) containing 500,000 cells per well were used for the stimulations, which were carried out for either 24 hours at 37 °C and 5% CO_2_ or 7 days (with 10% human pool serum added). Supernatants were gathered and kept at −20 °C until ELISA was used to measure the relevant cytokines. Specifically for the 24-hour stimulations, the stimulants used included poly I:C, LPS, imiquimod, IL-1a, HIV viral envelope, CMV and *S. pneumoniae*, and the cytokines measured included IL-1β, IL-1Ra, IL-6, IL-8, IL-10, MCP-1, MIP-1a and TNF. For the 7-day stimulations, the stimulants used included *E. coli*, *S. aureus*, *S. pneumoniae*, *M. tuberculosis*, *C. albicans* (conidia), *C. albicans* (hyphae) and PHA, and the cytokines measured included IL-5, IL-10, IL-17, IL-22 and IFNγ. A detailed explanation of the concentrations, manufacturers and strains can be found in the cohort publication.

Data processing was as follows. For the 24-hour experiment, samples from 1,742 participants were measured, of which 42 samples were excluded for being RPMI positive, defined as having concentrations of above 2× the lower limit of detection (LLOD) after RPMI stimulation in two out of TNF, IL-1b or IL-6. Outliers on PCA were defined as those with ±4 s.d. away from the mean of PC1 and/or PC2 (*n* = 13). Data from 1,687 participants were used in downstream analysis. For the 7-day experiment, samples from 1,744 participants were measured, of which 42 samples were excluded for being RPMI positive, and 20 outliers on PCA were removed as in the 24-hour experiments. Data from a total of 1,682 participants were used in downstream analysis. For the current analyses, only data from participants of European genetic ancestry were used.

#### Cell count

Whole blood samples were immunophenotyped using three flow cytometry panels, each with 17–20 markers, and custom-made tubes containing dry antibodies from DURA Innovations Technology (Beckman Coulter). Cells were collected in a 21-color, six-laser CytoFLEX-LX (Beckman Coulter) with CytExpert software 2.3. Daily instrument quality control and standardization were carried out with CytoFLEX Daily QC Fluorospheres (Beckman Coulter, cat. no. B53230), CytoFLEX Daily IR QC Fluorosphere Beads (Beckman Coulter, cat. no. C06147) and SPHERO Rainbow Calibration Particles, 6 peaks (Spherotech, cat. no. RCP-30-5A-6). The data analysis was performed using Kaluza version 2.1.2 and Cytobank Platform version 9.0 (Beckman Coulter).

### Multi-omics integration

All data modalities except genomics, namely epigenomics, transcriptomics, proteomics, metabolomics and cytokine production, were integrated by applying MOFA^14^. Each modality was preprocessed independently, and transcriptomics, metabolomics and cytokine production data were log normalized. To reduce the number of features in the epigenomics data (DNA methylation), the top 20,000 CpG probes with higher variance were selected. Each modality was corrected for age, sex and the institute in which the samples were collected by extracting the residuals of a linear model. Only samples with all modalities available in the discovery cohort were included. MOFA was run by applying the R function ‘run_mofa’ with default parameters, 30 factors and view scaling. Factors that correlated with the average of all features in any of the modalities with an absolute correlation above 0.6 were considered technical artifacts and were discarded for interpretation. Only factors that explained more than 1% of the variance across all modalities were considered for interpretation. This resulted in a total of 21 LFs.

### Association between LFs and other variables

The 21 LFs identified by running MOFA were tested for association with the immunological data, covariates and clinical variables present in the study.

**Extended Data Fig. 8:**
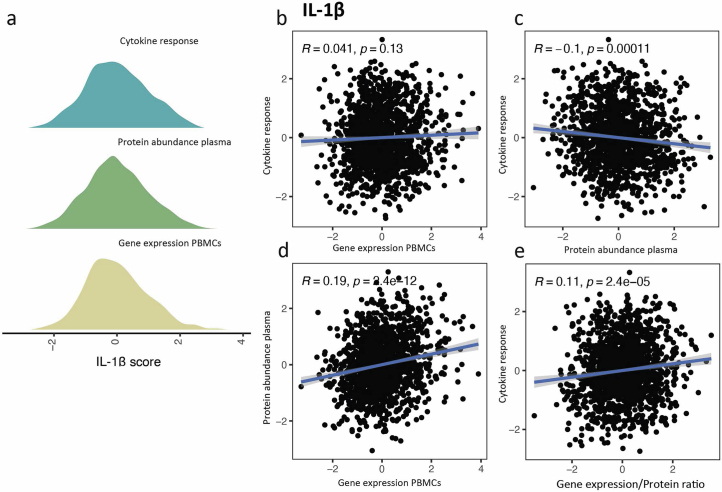
Multi-omics IL-1β scores. **a**, Distribution of IL-1β scores across three data layers. **b–e**, Spearman correlation of the IL-1β scores between each pair of data layers.

To associate with IL-1β levels, normalized and scaled protein, gene expression and cytokine values were used. A cytokine ‘IL-1β score’ was calculated by averaging IL-1β production among all 24-hour stimulations. Each of these three values was correlated to each of the 21 LFs using Pearson’s correlation, and the significance of the correlation was assessed by applying FDR correction. The inter-correlation between IL-1β scores was also calculated using Pearson’s correlation (Extended Data Fig. 8), including a measure of gene expression to protein levels ratio by calculating the difference between the normalized IL-1β levels between both.

A list of variables was assessed to associate LFs with covariates and clinical variables (Supplementary Data 2 and 3). Binary variables—for example, biological sex—were tested using the Wilcoxon rank-sum test. Categorical variables with more than two categories were tested by applying the Kruskal–Wallis test. Continuous variables were tested using Pearson’s correlation. All *P* values resulting from the multiple tests were corrected by applying FDR correction. The sign of the association was defined differently depending on the variable type: for binary variables, it was defined as the difference in the median between groups; for continuous variables, as the correlation sign; and for variables with multiple categories it was left as positive for visualization purposes.

### Molecular profiling of the multi-omic LFs

For each of the LFs, a ‘molecular profile’ was constructed to interpret the biological functions that it is capturing.

A set of features with significant weights was extracted for each factor and data modality. These significant weights were defined by standardizing the feature weights per factor and extracting features whose weight corresponded to a probability of 1% of having more extreme values, assuming a standard normal distribution.

Factors were tested for enrichment in different categories by running MOFA2’s R function ‘run_enrichment’, which is based on the principal component gene set enrichment method. Methylation, gene expression and protein weights were tested for enrichment against the gene sets defined by blood transcriptome modules^51^. Cytokine profile measures were tested for enrichment based on groupings made at the time of stimulation, the stimulant applied and the cytokine measured. All enrichments were run per sign—that is, separately for features with positive and negative weights—and with a minimum size of the feature set of 5.

### Multi-omic factor validation

**Extended Data Fig. 9:**
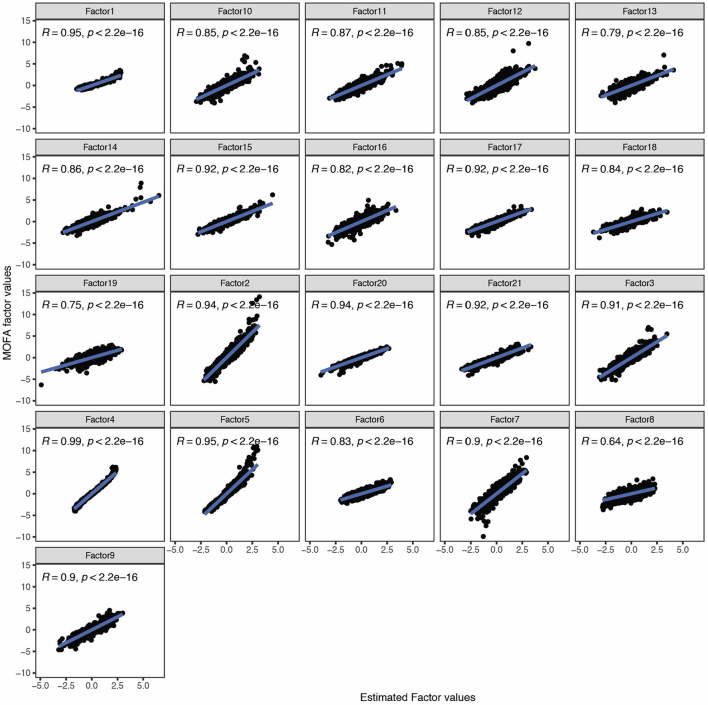
**Spearman correlation between factor values computed by MOFA and the values interpolated by multiplying the significant feature weights by the feature values**.

All LFs estimated by running MOFA in the discovery cohort were interpolated to the validation cohort. All data were normalized following the same steps as in the discovery cohort except for the correction for the institute of collection, as the validation cohort only includes samples that were collected in a different institute. Features with significant weights, as defined in the ‘Molecular profiling of the multi-omic LFs’ subsection, were extracted and used for the validation cohort, as this may reduce data overfitting. Factors were calculated by performing matrix multiplication between the preprocessed data and the significant weights. To test for the robustness of this method, the same approach was performed on the discovery cohort showing sufficient correlation (Extended Data Fig. 9).

### QTL mapping

Using both the discovery and validation cohorts, we performed QTL mapping for gene expression (*n*_disc_ = 1,048, *n*_val_ = 260), protein levels (*n*_disc_ = 1,064, *n*_val_ = 266) and metabolite levels (*n*_disc_ = 1,069, *n*_val_ = 267). For cytokine response QTL, each cytokine stimulation pair encompassed a different number of samples, ranging from 196 to 1,031 for the discovery cohort and from 41 to 260 for the validation cohort. We mapped the inverse-rank transformed values baseline omics (gene expression, proteins and metabolites) as well as cytokine response to the genotype data using a linear model that included age, sex, body mass index (BMI), seasonality, inclusion before the COVID pandemic, COVID vaccination and recruitment center. A sine and cosine wave with a period of 365.25 days was used to model seasonality. When combined, these two terms can create a sine wave with any phase and a yearly frequency^52^. QTL mapping was performed using the MatrixEQTL package^53^. Co-localization analyses were performed using the coloc R package^54^. We calculated study-wide *P* values by dividing GWs (5 × 10^−8^) by the number of effective tests. The number of effective tests was calculated following the formula described by Li et al.^55^, which takes into account the correlation matrix of the measurements.

For the comparison of the eQTL, pQTL and mQTL in the discovery cohort of PLHIV as opposed to healthy people, we used the public QTL databases GTEx^21^ and UKB-PPP^22^ as well as the mQTL from a healthy cohort^23^. We took the lead SNPs for all the GWs loci and looked for the same combination of SNP to trait in the corresponding full summary statistics, regardless of the significance of the association in the healthy cohort. We then estimated the concordance by considering the direction of effects as well as the correlation of the effects.

### Mendelian randomization

A systematic Mendelian randomization analysis was done using the R package TwoSampleMR^56^, with the inverse-variance weighted (IVW) method^57^ being the primary approach. For each exposure (gene expression, protein levels and metabolite levels), genetic instruments were carefully selected to ensure adherence to the assumptions. First, they were strongly associated (*P* < 1 × 10^−5^) with the exposure in the discovery cohort. They were then further validated, excluding any SNPs not nominal significant in the validation cohort. SNPs with MAF < 0.05 were filtered out. Lastly, SNPs associated with more than four other traits within the same exposure were considered too pleiotropic and were excluded. Next, stringent clumping was performed (*r*^2^ = 0.001, kb = 10,000) to ensure independent SNPs. Only if there were still at least three SNPs remaining, Mendelian randomization was performed. For Mendelian randomization with significant IVW (*P* < 0.05), sensitivity analyses were performed—horizontal pleiotropy, heterogeneity and leave-one-out. We performed the same Mendelian randomization in the other directions (bidirectional), and any results that had a nominal significant IVW result in the other direction, while also passing all the sensitivity checks, were excluded.

### Inflammasome score calculation

To estimate the activity of the inflammasome complex at both transcriptional and proteomic levels, a score was calculated per sample. For each sample, the inflammasome score was defined as the average of expression of all genes in the gene set using the covariate corrected values. The following gene sets were extracted from the Molecular Signatures Database (https://www.gsea-msigdb.org/gsea):

- *GOBP_POSITIVE_REGULATION_OF_INFLAMMASOME_MEDIATED_SIGNALING_PATHWAY.v2023.2.Hs*
- *REACTOME_INFLAMMASOMES.v2023.2.Hs*
- *GOCC_CANONICAL_INFLAMMASOME_COMPLEX.v2023.2.Hs*
- *REACTOME_THE_NLRP3_INFLAMMASOME.v2023.2.Hs*
- *GOCC_NLRP3_INFLAMMASOME_COMPLEX.v2023.2.Hs*

### Reporting summary

Further information on research design is available in the Nature Portfolio Reporting Summary linked to this article.

## Online content

Any methods, additional references, Nature Portfolio reporting summaries, source data, extended data, supplementary information, acknowledgements, peer review information; details of author contributions and competing interests; and statements of data and code availability are available at 10.1038/s41591-025-03887-1.

## Supplementary information

Reporting Summary

Supplementary Tables 1–6 Supplementary Data 1: Association between IL-1β scores and LF values. Supplementary Data 2: Association between LF values and clinical variables. Supplementary Data 3: Association between LF values and covariates. Supplementary Data 4: *cis*-eQTL with a significant opposite effect between PLHIV and healthy individuals. Only associations with an opposite effect in both eQTLGen and GTEx are shown. Supplementary Data 5: SWs QTL of LFs. Supplementary Data 6–26: Excel sheet containing the features—that is, genes, proteins, metabolites, methylation probes and cytokine stimulations—with a significant weight for each factor (defined in the Methods).

## Data Availability

All raw data are deposited in the Radboud Data Repository (10.34973/p96d-kz55). Summary statistics for QTL mapping and Mendelian randomization are available at https://lab-li.ciim-hannover.de/apps/hiv_xqtl_atlas.

## Code availability

All code used for this study is available at https://github.com/CiiM-Bioinformatics-group/HIV-integration.
