# Supplementary material for: Genetic and molecular landscape of comorbidities in people living with HIV
Source: Nat Med. 2025 Aug 20;31(10):3350–9. doi: 10.1038/s41591-025-03887-1 (PMC12532687; doi:10.1038/s41591-025-03887-1)
Supplement: Supplementary file 1 — Reporting Summary [file 41591_2025_3887_MOESM1_ESM.pdf]

nature portfolio

Corresponding author(s): Yang Li, André van der Ven

Last updated by author(s): Feb 4, 2025

Reporting Summary

Nature Portfolio wishes to improve the reproducibility of the work that we publish. This form provides structure for consistency and transparency in reporting. For further information on Nature Portfolio policies, see our Editorial Policies and the Editorial Policy Checklist.

Statistics

For all statistical analyses, confirm that the following items are present in the figure legend, table legend, main text, or Methods section.

n/a	Confirmed	
☐	☒	The exact sample size (*n*) for each experimental group/condition, given as a discrete number and unit of measurement
☐	☒	A statement on whether measurements were taken from distinct samples or whether the same sample was measured repeatedly
☐	☒	The statistical test(s) used AND whether they are one- or two-sided<br>*Only common tests should be described solely by name; describe more complex techniques in the Methods section.*
☐	☒	A description of all covariates tested
☐	☒	A description of any assumptions or corrections, such as tests of normality and adjustment for multiple comparisons
☐	☒	A full description of the statistical parameters including central tendency (e.g. means) or other basic estimates (e.g. regression coefficient) AND variation (e.g. standard deviation) or associated estimates of uncertainty (e.g. confidence intervals)
☐	☒	For null hypothesis testing, the test statistic (e.g. *F*, *t*, *r*) with confidence intervals, effect sizes, degrees of freedom and *P* value noted<br>*Give P values as exact values whenever suitable.*
☐	☒	For Bayesian analysis, information on the choice of priors and Markov chain Monte Carlo settings
☐	☒	For hierarchical and complex designs, identification of the appropriate level for tests and full reporting of outcomes
☐	☒	Estimates of effect sizes (e.g. Cohen's *d*, Pearson's *r*), indicating how they were calculated

Our web collection on statistics for biologists contains articles on many of the points above.

Software and code

Policy information about availability of computer code

Data collection	No software was used for data collection
Data analysis	PLINK v1.90, PLINK2 v2.00, snakemake 7.24, topr 2.0.2, MatrixEQTL 2.3, liftOver v2, TOPMed Imputation server (r2), minfi v4.2, DESeq2 1.36, MOFA2 v1.9.2, coloc v5.1, TwoSampleMR 0.5.6, CytExpert 2.3, Kaluza 2.1.2, Cytobank Platform V9.0<br>All code available at https://github.com/CiiM-Bioinformatics-group/HIV-integration

For manuscripts utilizing custom algorithms or software that are central to the research but not yet described in published literature, software must be made available to editors and reviewers. We strongly encourage code deposition in a community repository (e.g. GitHub). See the Nature Portfolio guidelines for submitting code & software for further information.

Data

Policy information about availability of data

All manuscripts must include a data availability statement. This statement should provide the following information, where applicable:

- Accession codes, unique identifiers, or web links for publicly available datasets
- A description of any restrictions on data availability
- For clinical datasets or third party data, please ensure that the statement adheres to our policy

The transcriptome and genotype data arAll raw data is deposited on the Radboud Data Repository (10.34973/p96d-kz55)
Summary statistics for QTL mapping and Mendelian Randomisation are available at https://lab-li.ciim-hannover.de/apps/hiv_xqtl_atlas.

Research involving human participants, their data, or biological material

Policy information about studies with human participants or human data. See also policy information about sex, gender (identity/presentation), and sexual orientation and race, ethnicity and racism.

Reporting on sex and gender	Throughout the manuscript we refer to the biological sex of the participants, as collected by self-reported questionnaires and confirmed with genotyping data. Further information on the 2000HIV study can be found in the original publication (Vos et al., Front. Immunol. 2022).
Reporting on race, ethnicity, or other socially relevant groupings	The participants included were part of the 2000HIV study. Only participants of self-reported and genetically confirmed European genetic ancestry were included to mitigate the effect of population stratification on genetic associations. Further information on the 2000HIV study can be found in the original publication (Vos et al., Front. Immunol. 2022).
Population characteristics	The participants in the study are virally suppressed people living with HIV in the Netherlands. A detailed description of the cohort can be found in Vos et al., Front. Immunol. 2022. The cohort has a 89% of male participants and a median age of 54 years, and has been living with HIV a median of more than 10 years.
Recruitment	Participants were recruited from October 2019 until October 2021. The 2000HIV study is made up of the discovery cohort and the validation cohort. Participants in the discovery cohort were recruited in three specialized Dutch HIV treatment centers, two university medical centers and one large general hospital (Radboudumc Nijmegen, Erasmus MC Rotterdam, and OLVG Amsterdam). Participants in the validation cohort were recruited in a separate medical center, a large general hospital (Elisabeth-TweeSteden Ziekenhuis Tilburg). Further information on the 2000HIV study can be found in the original publication (Vos et al., Front. Immunol. 2022).
Ethics oversight	The 2000HIV study protocol was approved by an accredited medical research ethics committee, the Independent Review Board Nijmegen (NL68056.091.81) and published at clinicaltrials.gov (ID: NCT03994835). Written informed consent was received from participants prior to inclusion in the study. All experiments with human samples were conducted according to the principles expressed in the Declaration of Helsinki.

Note that full information on the approval of the study protocol must also be provided in the manuscript.

Field-specific reporting

Please select the one below that is the best fit for your research. If you are not sure, read the appropriate sections before making your selection.

☒ Life sciences ☐ Behavioural & social sciences ☐ Ecological, evolutionary & environmental sciences

For a reference copy of the document with all sections, see nature.com/documents/nr-reporting-summary-flat.pdf

Life sciences study design

All studies must disclose on these points even when the disclosure is negative.

Sample size	Earlier studies in the Human Functional Genomics Project provided insights on the role of genetic and environmental factors in inter-individual differences in immune function. Cohorts of 250 to 500 individuals, matched with patient-specific cohorts aided in understanding the diversity in immune responses. In People Living with HIV, a prior study with 200 individuals led to the identification of relevant pathways and markers in this group, as well as targeted genetic and omics factors. In the 2000HIV study, increasing the sample size significantly will allow to identify more robust associations and relevant markers, also allowing to increase the dimensionality of the data to measure a broad number of omics readouts.
Data exclusions	Exclusion criteria were: no informed consent, insufficient communication because of language barriers or other problems, current pregnancy, detectable viral hepatitis B or C DNA by polymerase chain reaction (PCR) or signs of any current acute infection
Replication	A validation cohort was recruited in a different recruitment location, already described in "Recruitment". All relevant multi-omic factors were tested for replication in the validation cohort. In Mendelian Randomisation estimates, the genetic variants used were tested for replication in the validation cohort.

Randomization	The 2000HIV study is an observational and longitudinal study, therefore, randomisation was not necessary.
Blinding	No randomization was performed and therefore no blinding was necessary.

Reporting for specific materials, systems and methods

We require information from authors about some types of materials, experimental systems and methods used in many studies. Here, indicate whether each material, system or method listed is relevant to your study. If you are not sure if a list item applies to your research, read the appropriate section before selecting a response.

Materials & experimental systems

n/a	Involved in the study
☒	☐ Antibodies
☒	☐ Eukaryotic cell lines
☒	☐ Palaeontology and archaeology
☒	☐ Animals and other organisms
☐	☒ Clinical data
☒	☐ Dual use research of concern
☒	☐ Plants

Methods

n/a	Involved in the study
☒	☐ ChIP-seq
☒	☐ Flow cytometry
☒	☐ MRI-based neuroimaging

Clinical data

Policy information about clinical studies

All manuscripts should comply with the ICMJE guidelines for publication of clinical research and a completed CONSORT checklist must be included with all submissions.

Clinical trial registration: clinicaltrials.gov, ID: NCT03994835

Study protocol: A full study protocol can be found in Vos et al., Front. Immunol. 2022.

Data collection: Participants were recruited from October 2019 until October 2021. The 2000HIV study is made up of the discovery cohort and the validation cohort. Participants in the discovery cohort were recruited in three specialized Dutch HIV treatment centers, two university medical centers and one large general hospital (Radboudumc Nijmegen, Erasmus MC Rotterdam, and OLVG Amsterdam). Participants in the validation cohort were recruited in a separate medical center, a large general hospital (Elisabeth-TweeSteden Ziekenhuis Tilburg). Further information on the 2000HIV study can be found in the original publication (Vos et al., Front. Immunol. 2022).

Outcomes:

Primary Objectives

Identify a set of candidate biomarkers that correlate with particular non-AIDS-related comorbidities Unravel biological processes associated with extreme HIV clinical phenotypes.
Find therapeutic targets to identify novel assets or for repurposing of clinical phase assets from other disease areas for HIV.
Secondary Objectives

Evaluate potential relationship of host/immune profiles on efficacy, safety, and tolerability of standard care regimens. Evaluate the contribution of age, sex, and genetics in host-immune profiles that are:

distinct to HIV infection relative to controls in other cohorts;
associated with non-AIDS-related comorbidities in HIV infection relative to non-HIV chronic disease.

In the current manuscript we evaluated the first primary objective by identifying biomarkers using multi-omics factor analysis and Mendelian Randomisation, and the first secondary objective by assessing whether the genetic regulation of omics measurements and immune function correlated with those found in individuals without HIV.

Plants

Seed stocks	n/a
Novel plant genotypes	n/a
Authentication	n/a
